# “…in the middle of nowhere…” Access to, and quality of, services for autistic adults from parents’ perspectives: a qualitative study

**DOI:** 10.3389/fpsyt.2024.1279094

**Published:** 2024-02-26

**Authors:** Vivien Németh, Miklós Győri, Bea Ehmann, Márta Völgyesi-Molnár, Krisztina Stefanik

**Affiliations:** ^1^ Bárczi Gusztáv Faculty of Special Needs Education, Institute of Special Needs Education for People with Atypical Behavior and Cognition, Eötvös Loránd University (ELTE), Budapest, Hungary; ^2^ Hungarian Academy of Sciences (HAS) – Eötvös Loránd University (ELTE) ‘Autism in Education’ Research Group, Budapest, Hungary; ^3^ Faculty of Education and Psychology, Doctoral School of Education, Eötvös Loránd University (ELTE), Budapest, Hungary; ^4^ HUN-REN Research Centre for Natural Sciences, Institute of Cognitive Neuroscience and Psychology, Budapest, Hungary

**Keywords:** autism, adulthood, parental perspective, access to services, quality of services

## Abstract

**Introduction:**

Adequate education, employment, and services for autistic individuals contribute significantly to their and their parents’ quality of life. Services and support for adults are dramatically more limited than those for children. The main purpose of this study was to explore how parents perceive factors supporting/hindering access to services, and how they assess the quality of services.

**Methods:**

Qualitative data provided by 12 parents via a semi-structured interview with a broader focus on parental quality of life and its factors were analyzed. Their autistic children were between 20 and 34 years of age. A thematic analysis was performed on parts of the narratives on their adult periods of life.

**Results:**

A complex pattern of parental perception of supportive and hampering factors influencing access to services unfolded. The sparsity of services/activities and reliable information on them made the space for autonomous decisions on service take highly limited. Parents have modest expectations on quality of services, evaluating them along two key aspects: a safe, positive atmosphere, and communication between parents and professionals. Other aspects of individualized autism-specific support were not or just rarely mentioned.

**Discussion:**

Parents perceive themselves as investing a lot of effort and resources in getting some form of regular service and/or activity for their adult child. However, these parental efforts often fail, their child becoming inactive, and dependent on their presence. This suggests system-level problems with services for autistic adults in Hungary, with literature showing it is not specific to this country.

## 1 Introduction

Autism is a diverse set of pathways of human development, with specific challenges in socio-communication and social reciprocity, and with stereotyped, repetitive patterns of interest and behavior (1). Patterns of strengths and difficulties are highly variable along several dimensions, age being a significant one (2, 3). Appropriate intensity and quality of autism-specific support and access to services can improve the quality of life of the autistic individuals, at any age (4–7). As autistic young people leave school, however, support often decreases, with fewer services being available for adults (8–13). Parents’ quality of life and psychological well-being are strongly affected by their adult child's access to regular services (e.g., education) and activities (e.g., employment). Systematically designed and implemented services, which offer a foreseeable pathway, provide the feel of safety for the parents. The lack of systematic and regular support, however, leads to a reduced parental sense of competence, elevated level of parental stress, as well as to the experience of being lost (14, 15). An important question is therefore how parents perceive the factors that support or hinder the access to services and how they assess the quality of available services.

Little research is yet available on the determinants of access to services for adults. These studies come from different countries and service systems, but four groups of factors seem to be important across these. Place of residence is a key socio-economic determinant, as geographically uneven service availability strongly influences access to appropriate support and care (16–19). Secondly, family resources are also a crucial factor, as often only (expensive) privately paid services are available for adults. Managing access and traveling to services can also be a significant burden (8, 9, 16, 19, 20). Thirdly, individual characteristics are a factor of access, as services for adults without co-occuring intellectual disability (ID) and complex communication needs (CCN) or with challenging behaviors are especially sparse (9, 19, 21). Finally, a very important factor is the parents’ ability to gather information on services and activities, by mobilizing their own resources (8, 16, 22–24). Parents seem to base their service-related decisions mainly on the opinions of family members, acquaintances, parent organizations, and on information provided by the media and the internet, rather than on professional resources (10, 25). The collected information is very uneven in quality, often insufficient in quantity, and difficult to understand (8, 9).

Several factors determine families’ decisions on, and evaluation of, available services. Parental perspective often shows that families have no real choice, as there are no or just very few services available (8, 9, 16, 23, 24, 26), which leads to a decrease in parental well-being (14). Where choice is available, both parental decisions on, and satisfaction with, services are determined by *(a)* whether there is collaboration and mutual communication between parents and professionals (8, 23, 27); *(b)* whether the parent perceives the client group size appropriate; *(c)* whether the professionals are adequately trained and experienced (8, 27); and *(d)* whether autism-specific programs and methodologies are available (8, 27). Parental evaluation of *(e)* the physical environment is less important in the choice of service but more important in the perception of its quality (27). Other factors influencing parents’ perceptions of the quality of service are *(f)* the alignment of the social environment with individual abilities and needs (8, 27); *(g)* the psychological well-being of the autistic adult (8, 27); *(h)* the adult’s detectable development (8, 23, 27).

In Hungary, according to current legislation, it is the state’s responsibility to create and maintain adequate services for autistic individuals. However, the state-provided system of support for autistic adults is not integrated but distributed between public education, higher education, health and social care systems. To date, only two quantitative parent surveys have been conducted to explore the situation of autistic adults in Hungary (28, 29). Their results are worrying: most adults do not receive autism-specific support, and approximately 50% of them are not employed and/or supported by educational services (29).

The focus of this qualitative study is on autism-related support, education, housing, and employment services for autistic adults, from the parents’ perspective. That is, we focus on factors which influence parental quality of life. Our specific aims are *(a)* to explore the factors that parents perceive as hindering or facilitating access to services and activities, and *(b)* to identify the factors that influence parents’ perceptions of the quality of services.

## 2 Methods

### 2.1 Participants

Parents were recruited from the sample of a previous quantitative study (29–31). From among the volunteering 323 parents, we selected a total of 32 participants randomly, by using a layered sampling with niches along *(a)* the age, *(b)* the individual profile of intellectual and language abilities, *(c)* the type of place of residence, and *(d)* the maternal level of education. In the present study, we analyzed all the interviews from parents of autistic adults (n=12; age range 20 – 32 years; 11 mothers and 1 father). All autistic individuals had an autism spectrum disorder diagnosis, which, with one exception, they received in their childhood (between ages of 2 and 10 years). In one case, the diagnosis was received at the age of 23. Six of the autistic individuals did not have a co-occuring ID and CCN, while six of them had ID and CCN. **Appendix 1** offers more data on the respondents and their autistic children.

### 2.2 Data collection

In designing the interview and the setting for its administration we followed the recommendations of Cridland et al. (2015) (32). The main focus of the investigator-based interview was on understanding better parental quality of life in connection to the services received by autistic individuals. Partial foci were on *(a)* the current life circumstances of the autistic individuals and the parent/family supporting them; *(b)* reconstructing their personal life history; understanding parents’ experiences about *(c)* their decisions on services and institutions, and about *(d)* the educational and other services received by their children. Interviewers were two professionals with both clinical and interviewing expertise.

A minimum of 10 topics were to be discussed in the interview (see **Appendix 2**). The length of the interview depended on the number of services used by the autistic adults, as each service was explored separately, along the same questions. Administering the interviews took 75-120 mins.

Interviews were administered in locations easily accessible to the respondent parents, in institutions/rooms appropriate for personal discussions but not related to any service received by the autistic individual or the family.

### 2.3 Analysis

All data processing and analyses were done on literal transcriptions of the audio recordings of the interviews by the first and last authors of this paper. First, a 2-phase data preparation was done on these, using Microsoft Excel. In phase 1, 2 researchers coded the entire interview texts, independently of each other, along the following pre-defined content categories: the autistic individual; the family; service/intervention; other key persons and settings. Sentence-level text units were coded. Inter-coder reliability was above 92% on all codes in the entire data set. In phase 2, we reconstructed the educational/employment/autism-related service career for each autistic individual.

Thematic analysis was then performed on parts of interview texts relevant to the adult periods (>18 years of age) of the autistic individuals. This was done in the six steps of the analysis framework by Braun and Clarke (2006) (33), using the ATLAS.ti software. Open coding was performed on the relevant interview parts, along with 3 foci derived from our research questions: (a) form of services (support) received; (b) factors supporting or hampering the access to these services; (c) aspects of evaluating these services by the parents. The codes aligning with these foci were examined on their contents and then grouped into subthemes and broader themes.

## 3 Results

### 3.1 A key background of results: where are autistic adults?

In this section, we briefly review the services and supports the autistic individuals in our study received through their adulthood. This provides an important context for interpreting our further findings.

Autistic adults’ educational histories were heterogeneous: four young adults studied in mainstream high schools, eight studied in special schools, and two participated in vocational education. Two of them went to higher education but they did not complete their studies. *“[The university] was such a big burden on him that he could hardly, hardly bear it; and the examination period was a real torment” (P11_26).*

Two young adults visited a day care center regularly, at the time of the interviews, one of them had regular swimming training sessions, too. *“As I see, [my daughter is] happy, because the most important thing in her life is swimming, and we somehow found it when she was still a small kid, and now we are going to training sessions regularly, and this fills up her life and her time very much; we go to competitions, too” (P2_23).*

Only three adults had any short work experience, and only one was employed at the time of the interviews, in a sheltered employment. A lack of employment for their adult child had a deep impact on the parents too, as they had to stay at home with them. *“When [my son] turned 18, then school stopped for him. We were looking for sort of day care centers. Everyone was looking for these, from the school, too, everyone. So, I have been staying at home since then (…)” (P5_28).* Only one adult in the study lived in a residential home, all the others lived with their parents, at the time of the interviews.

**Figure 1:**
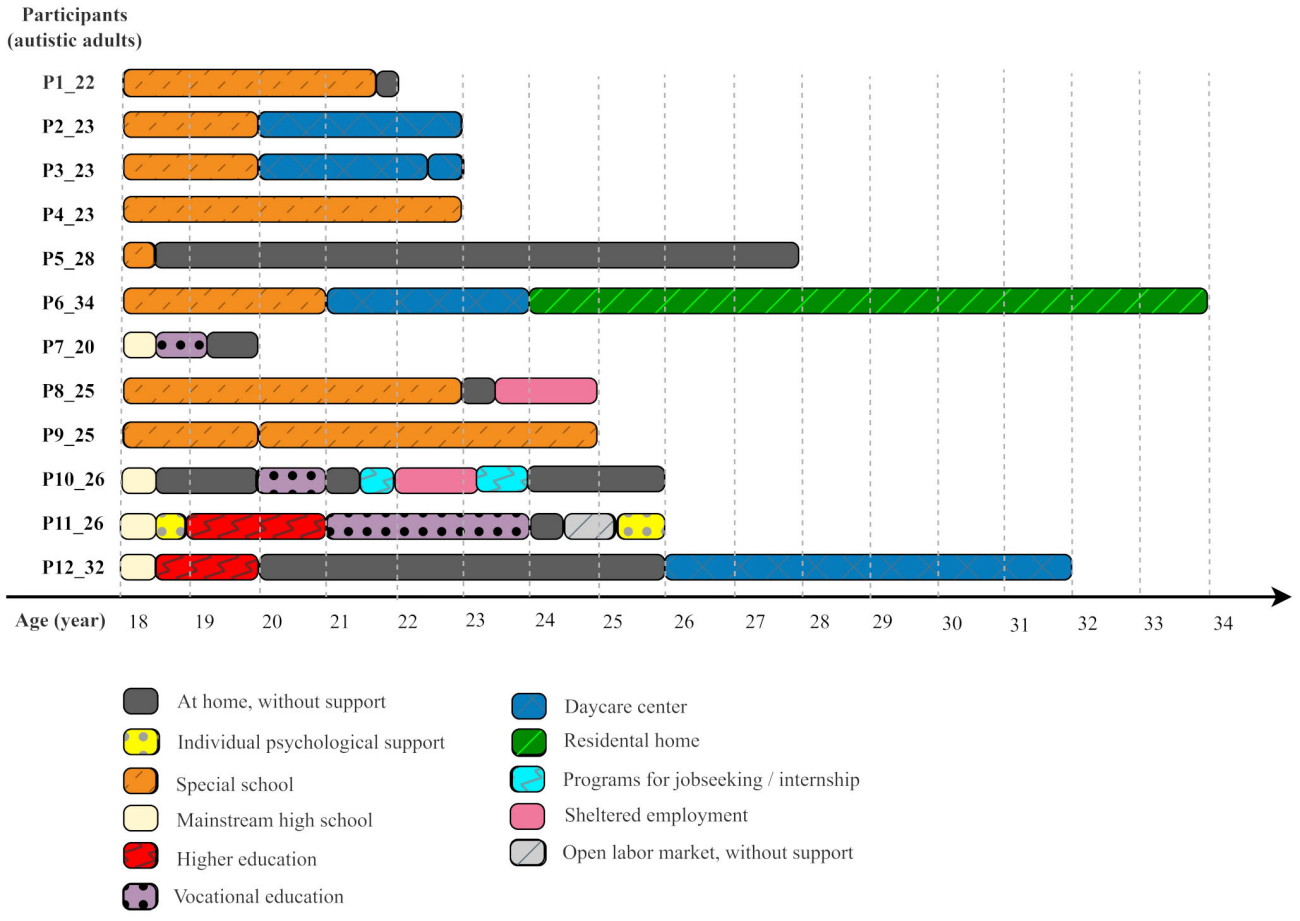
Individual life paths services and activities above the age of 18 years.

The autistic adults in the focus of our study often became isolated and inactive – irrespective of the level of their intellectual and language skills, individual profile of abilities, and their former school career (**Figure 1**.). *“That, now, if he is able or unable to work, that’s one thing. But he is living his life, anyhow, and he should not do it this way, just behind the walls of his room” (P12_32).*

### 3.2 Factors supporting and impeding access to services and activities

**Figure 2:**
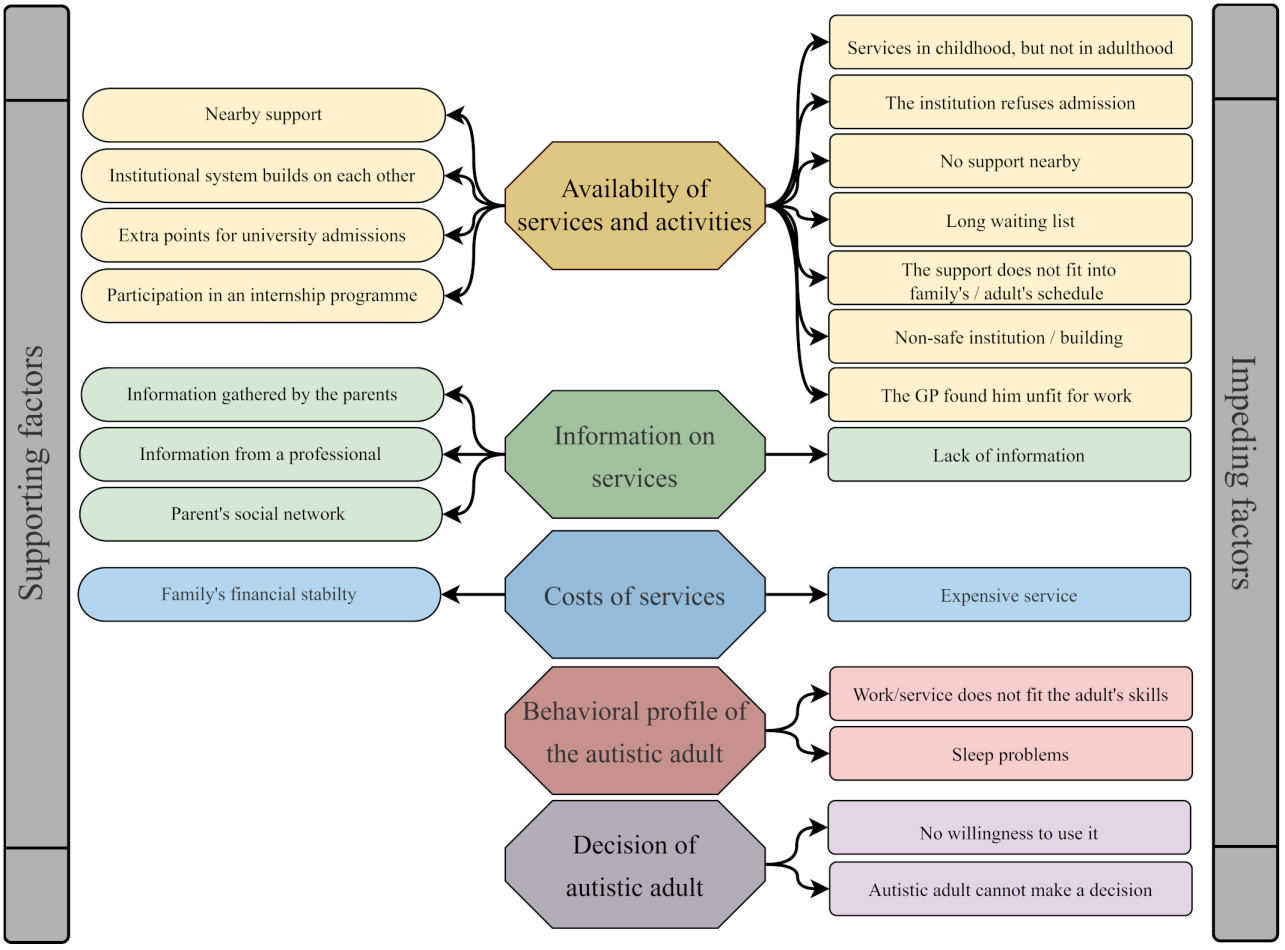
Themes and subthemes on access to services and activities.

Factors supporting and impeding access to services and activities were mentioned in all parental interviews. In the following, we focus on the five broader themes, briefly indicating the sub-themes that make them up (see **Figure 2**). We illustrate the key findings by quotes (see **Appendix 3** for further quotes).

The most dominant factor appears to be *the availability of services and activities* (4 supporting and 7 impeding factors). This interacts with all further themes. The most prominent experience of the parents is the lack of autism-specific support for adults. A mother living in the countryside described their situation as follows: *“And, after all these institutional services stopped, we are now standing here in the middle of nowhere, and there is nothing more, no one calls as like dear parent, please come; if anything happens to exist at all, it is there [in the capital city]” (P3_23).* Autism-related services are either non-existent or just sporadic in the vicinity of families; waiting lists for existing services are very long. Accessibility of services available further away is limited not only by longer travel time and higher travel costs but also by their timing: services are provided almost exclusively on weekdays, interfering with parents’ working times. Transparent pathways within the support systems improve parents’ psychological well-being. One parent finds it reassuring when special services for various age groups are accessible in the same institution – this gives them foresight about potential services accessible later for adults, even in childhood: *“I’ve never had to worry because I’ve always known that one institution builds on another.” (P4_23).* It is also experienced as a supporting factor by parents that the autism diagnosis entails a special advantage in the university admission procedure. Also, internship programs can help in getting paid employment later on.

*Information on services is a crucial* theme, too (with 3 supporting and 1 impeding factor). Parents emphasized most often that they themselves had to go after everything, under their own power. They perceive easily available information as being very limited. Collecting more, however, takes a lot of energy from their part: *“… the main experience is just coming up against brick walls, and if you want to be sure, you have to do it yourself [information gathering]. But I have had enough of it…” (P10_26)*. On the other hand, the possibility of getting useful knowledge from professionals, and from their own social network represents a supporting factor for parents.

The *costs of services* have also emerged as a strong determinant of access to services in the parental narratives (both as a supporting and an impeding factor). In one of the mother’s words, speaking about residential homes: *“(…) well, these are kind of horrible prices, but even so, waiting lists go up to hundreds-long, meaning years” (P3_23).*

Factors influencing access to services include the *behavioral and ability profile of the autistic individual*, too. The two connected subthemes are impeding ones. In five parents’ perceptions the accessible services do not meet their children’s needs. The mother of a young adult commented on this, after her child dropped out of higher education: *“He then went to another company, I’d call it a sheltered employment; they said he was so slow. He had to assemble curtain clips, but that didn’t engage his attention. So, the problem with such employments is that he is, in fact, I think, too clever for assembling curtain clips” (P12_32).*

The theme “the decision of the autistic adult” emerged in relation to two adults only, solely as a barrier. One of them had severe difficulties in making any important decision. The mother of the other adult explained the refusal of the special service as follows: *“(…) up to the present day, he keeps saying that he would like to work as, so to say, as a normal person, not having a stigma on him” (P11_26).*

As the risk of inactivity is very high, parents can only focus on one main goal: to have *some* service for their children. Often, therefore, there is no space at all for a real decision with real options, and for the person’s or the parents’ own preferences to play any role.

### 3.3 The activities in the parents’ perceptions

**Figure 3:**
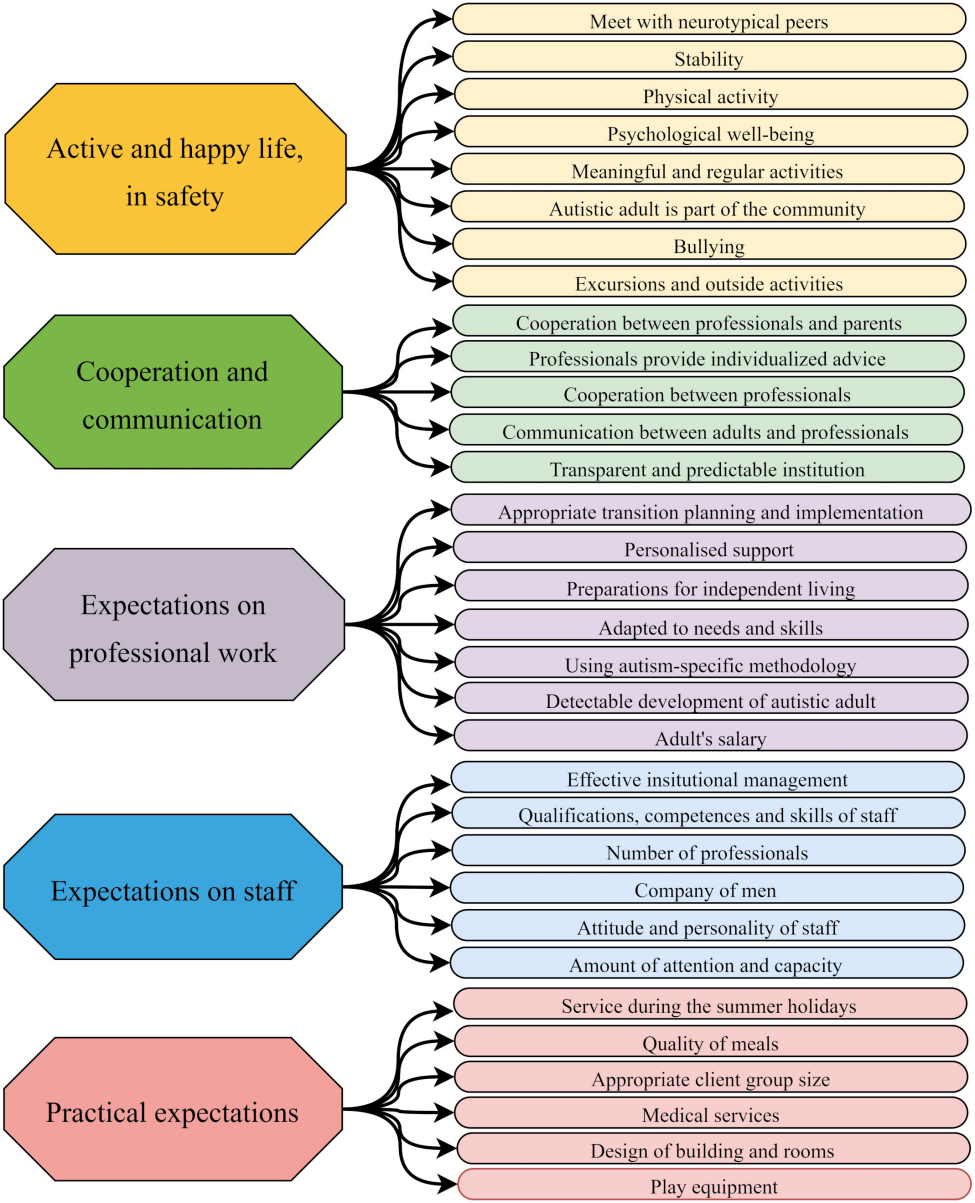
Aspects of parents’ evaluation of the quality of services.

Five broader themes emerged along parental evaluation of services/activities (**Figure 3**; see **Appendix 4** for further quotes). These can influence parents’ opinions both positively and negatively.

It is naturally of key importance for parents that their children live an *active and happy life, in safety* (8 subthemes are connected to this broad theme). “*I’d only like him to be happy and enjoying what he does. (…) To have him feeling important, feeling he is suitable for that position. And, above all, what is important for everyone, is getting positive acknowledgement. Well, that’s all” (P10_26).* Importantly, autistic adults should be able to spend their time doing meaningful activities. One of the parents said about a day care center: “*That is a superb institution. Well, they really keep all his time engaged, there is no idle time to be just sitting and staring, they keep them moving, exercising, running, doing aerobic activities, making music, doing housework, handicrafts, and there is even developmental intervention” (P2_23).* It is also important for the parents that their child become part of a community, but they cannot achieve this without supporting services: “*I am unable to create a community around my child, just with help from relatives and friends; so, our only community is the school” (P4_23).*

From the parents’ perspective, *cooperation and communication* form a dominant theme (embraces 5 subthemes). The cooperation between parents, institutions, and professionals is an important factor in assessing the quality of the services. *“There is a case manager, she helps us as much as possible, even in personal matters which are not among her responsibilities, but when I call her, she takes care of it (…); all in all, she really looks after us” (P12_32).*

The broad theme of *expectations on professional work* (embraces 7 subthemes) includes a range of parental considerations. The importance of using autism-specific methods emerged only in the narratives of parents who are themselves professionals, or whose children have received high-quality autism-specific services before. Some parents emphasized the importance of planning and implementing the transitions between institutions, too. Two parental narratives referred to salary in connection to employment of their child. These parents emphasized that it is not the amount of salary that is important for them, but its meaning to their children, and that it makes them feel appreciated. Two other factors seem to play a key role in shaping parents’ opinions of professional work: one is the match between the support and the needs and abilities of the autistic adult, and the other is for the development to be perceivable, especially in relation to skills relevant to independent life. These influence parental sense of competence to a large extent. *“I am in a difficult situation because I am living with him. And, therefore, I may take over a bunch of things from him, which I know I should not (…). And having a professional here would be good. To prepare him for life itself, and for reacting to certain situations” (P10_26).*

The broad theme of *expectations on staff* embraces 6 subthemes. Among these, the most prominent seem to be the expectations of the professionals’ attitudes and personality. *“Professionals are totally superbeings. Well, all of them are nice, cute, attending [to my child] totally” (P2_23).* The qualifications and aptitude of the professionals emerge with similar importance to these*. “There is not at all any [autism-specific support], not to the present day, any. That’s why he is still unable to talk about his sorrows, his grievances. If there were any [professional] there, they’d use a daily schedule, some communication aid; I am trying these here at home, but, unfortunately, I cannot teach him these here at home” (P6_34).* Further emerging subthemes are the number of professionals, and the presence of male professionals in the dominantly female profession, as well as the effective management of the service institutions.

The broad theme of *practical expectations* embraces 6 subthemes. Most of these are related to the conditions and equipment of the institutions. As for the layout and conditions of the service rooms, parents focus on their functional appropriateness for service provision, and much less on the quality or aesthetics of the rooms and equipment*. “(…) they are not prepared for autistic adults. The gym is far too small, there are no toilets for adults, the building is not fit for adult-sized autistic people” (P4_23).* Additional services beyond the most fundamental ones, which might be important for parents were mentioned just sporadically. They mentioned some positive examples: availability of medical services within the institution, availability of supervision in the summer breaks, physical exercise options, quality of the food provided, and outdoor programs.

## 4 Discussion

Access to appropriate autism-specific services has a significant impact on the quality of life of autistic adults and their parents (6, 34, 35). Based on a thematic analysis of the narratives of parents in our study, it appears that social participation of autistic adults is low, with limited access to appropriate services and activities. As a result, adults and their parents often become isolated, inactive and/or overwhelmed, and their resources are depleted. These, then, lead to fatigue in the parents and a deterioration in their psychological well-being. This is not a phenomenon specific to Hungary, similar systemic problems are highlighted in the international literature (14, 16, 26, 36).

A dominant and distressing perception of the parents is that they do not see any perspective that suits their child’s individual ability profile after leaving public education. There is a severe shortage in services for autistic adults, and there are no real choices for the families. This is particularly the case for parents of adults without ID. These findings are in line with the results of international studies (13, 37).

Parents have also seen promising initiatives in housing, employment and higher education (for example, in Hungary, ASD diagnosis means extra points for university admission). However, these supportive factors do not guarantee a long-term solution or success, and the outcome is often frustration. The drop-out of young autistic adults from higher education, and/or their failure to find paid employment are sources of stress for parents too, and seem to be important steps towards long-term isolation and dependence on the parents’ presence at home (38).

On the other hand, if parents can anticipate earlier in their child’s childhood which services will be available later, it can improve their psychological well-being. However, this requires not only the establishment and maintenance of a range of services for autistic adults, but also the provision of reliable, understandable and easily accessible information. The phenomenon of parents having to mobilize their own resources to gather information with great difficulty is also not specific to Hungary (see for example (9, 39)).

Hungarian parents seem to have modest expectations of the quality of services, evaluating it on two key aspects: a safe, positive atmosphere and communication between parents and professionals. Other aspects of autism-specific support tailored to individual needs are missing from the interviews or are just rarely mentioned, although they are of great importance for the quality of life of autistic adults and their families (35, 40). In contrast, in qualitative studies from the US, for example (25, 41), parents mention autism-specific components of services as of key importance for them; specifically, support for socio-communication and behavior organization, and the training of professionals. The need for more diverse forms of support (e.g. online services, community-based support) does not appear in the narratives of Hungarian parents. This may be because it is very difficult to get access to a service or activity, so there is no opportunity to consider the quality of these. This seems to be the reason why parents rarely consider, if at all the possibility that their autistic adult children could make an informed decision about what they want to participate in.

Altogether, parents of autistic adults in Hungary perceive themselves as investing a lot of personal effort and family resources in getting some form of regular support service and/or activity for their adult child. In spite of these efforts, they often see the outcomes as a failure, seeing their child becoming socially isolated, inactive, and dependent on their presence, for the long turn. This reduces parental sense of competence, too, which is an important factor in their psychological well-being (14).

Our results provide further evidence that to improve the quality of life of autistic adults and their parents a systematic improvement of the service system is needed, based on input from research. Although a growing number of studies focus on supporting autistic adults internationally (42, 43), this trend cannot yet be seen in Hungary. Autistic adults and their parents should have a voice in the process of designing and improving services systematically. In this study, we aimed at presenting the parents’ perspective, but it is essential for further research to focus autistic adults in Hungary, too.

Methodological limitations of the present study include the small sample size and the fact that the parent interviews were analyzed along only two main foci. We also need to treat the results with caution because the children of the parents in our sample are young adults, allowing for the possibility that parents of older autistic adults may have different relevant experiences, as highlighted in previous studies (44–46). Further refined analyses and mixed-mode research are needed to see more precisely which factors related to the service system affect the quality of life of autistic adults and their parents. Importantly, although our key results are in line with findings from the international literature, the present study does not allow a comparison of the severity of the difficulties parents face in Hungary vs. in other countries.

## Data availability statement

The raw data supporting the conclusions of this article will be made available by the authors, without undue reservation.

## Ethics statement

The studies involving humans were approved by The Scientific and Research Ethics Committee of the ‘Bárczi Gusztáv’ Faculty of Special Needs Education of ELTE University, Budapest, Hungary. The studies were conducted in accordance with the local legislation and institutional requirements. The participants provided their written informed consent to participate in this study.

## Author contributions

VN: Conceptualization, Data curation, Formal analysis, Methodology, Project administration, Resources, Visualization, Writing – original draft. MG: Conceptualization, Funding acquisition, Methodology, Project administration, Writing – original draft. BE: Methodology, Writing – review & editing. MV-M: Conceptualization, Data curation, Funding acquisition, Methodology, Project administration, Writing – review & editing. KS: Conceptualization, Data curation, Formal analysis, Funding acquisition, Methodology, Project administration, Supervision, Visualization, Writing – original draft.
