# Supplementary material for: “…in the middle of nowhere…” Access to, and quality of, services for autistic adults from parents’ perspectives: a qualitative study
Source: Front Psychiatry. 2024 Feb 26;15:1279094. doi: 10.3389/fpsyt.2024.1279094 (PMC10946251; doi:10.3389/fpsyt.2024.1279094)
Supplement: Supplementary file 1 [file Table_1.docx]

Supplementary Material

| Participant | Mother’s education level | Housing | | Age and gender of adults with ASD | Age (y) at ASD diagnosis | ID | CCN | Associated diagnosis |
| --- | --- | --- | --- | --- | --- | --- | --- | --- |
| P1 (mother) | high school | | village | 22  Male | 8 | yes | yes | - |
| P2 (mother) | university | | town | 23  Female | 10 | yes | yes | - |
| P3 (mother) | university | | town | 23  Male | 2 | no | yes | - |
| P4 (mother) | high school | | town | 23  Male | 2,5 | no | yes | anxiety |
| P5 (mother) | high school | | capital | 28  Male | 3 | no | yes | - |
| P6 (mother) | university | | town | 34  Male | 3 or 4 | no | yes | - |
| P7 (mother) | university | | village | 20  Male | 4 | no | no | depression |
| P8 (father) | university | | town | 25  Male | 23 | yes | no | scoliosis, heart-disease |
| P9 (mother) | high school | | capital | 25  Male | 6 | no | no | - |
| P10 (mother) | high school | | town | 26  Male | 3,5 | no | no | - |
| P11 (mother) | university | | capital | 26  Male | 8 | no | no | lactase acidosis, kidney disease |
| P12 (mother) | high school | | capital | 32  Male | 8 | no | no | allergy (to pollen) |

ID: intellectual disability; CCN: complex communication needs

Table 1. Characteristics of parents and their adult children
