# Supplementary material for: “…in the middle of nowhere…” Access to, and quality of, services for autistic adults from parents’ perspectives: a qualitative study
Source: Front Psychiatry. 2024 Feb 26;15:1279094. doi: 10.3389/fpsyt.2024.1279094 (PMC10946251; doi:10.3389/fpsyt.2024.1279094)
Supplement: Supplementary file 2 [file Table_2.docx]

Supplementary Material

|  | Topics of the interview |
| --- | --- |
| 1. | Background information and current situation, social context |
| 2. | The autistic child: the current situation |
| 3. | The story and trajectory of parent’s life |
| 4. | The life story of the autistic child/adult |
| 5. | The expected future |
| 6. | Overview of the services: institutional pathway |
| 7. | Overview of the services: outside of institutions |
| 8. | The diagnosis/diagnoses |
| 9. | What could make the situation better (in general)? |
| 10. | Closing: topics important to the parent but haven’t discussed yet; discussion of any further questions from the parent |

Table 2. Structure of the semi-structured interview
