# Supplementary material for: “…in the middle of nowhere…” Access to, and quality of, services for autistic adults from parents’ perspectives: a qualitative study
Source: Front Psychiatry. 2024 Feb 26;15:1279094. doi: 10.3389/fpsyt.2024.1279094 (PMC10946251; doi:10.3389/fpsyt.2024.1279094)
Supplement: Supplementary file 3 [file Table_3.docx]

Supplementary Material

| Broader themes | +/- | Example quotes |
| --- | --- | --- |
| Availability of services and activities | + | *“(...) I think I was admitted to the university, because there were those extra 40 points, and**, by the way, they came in handy for those subjects, (...) I almost fainted for the happiness I felt.” (P11_26) [subtheme: extra points for university admissions]* |
|  | - | *“He completed the school, and we thought it would be* *pretty simple to get some services after that – well it was not, at all.” (P8_25) [subtheme: services in childhood, but not in adulthood]* |
| Information on services | + | *“[It is a help from the professionals], that there are topics which they take off from my shoulders;* *so to say, I don’t have to noodle these around (...), they just find the alternatives.” (P8_25) [subtheme: information from a professional]* |
|  | - | *“No one can help us [in collecting information], as a matter of fact, there isn’t around any professional with us for helping us, since he is 20 now and got out from any institutional service provision.” (P7_20) [subtheme: lack of information]* |
| Costs of services | + | *“I started to collect money heavily [for the services]. (...) That is, I did not just eventually changed job (...).” (P10_26) [subtheme: family's financial stability]* |
|  | - | *“(...) We can just make ends meet; I just pay for it [the individual psychological support] and then that’s all about my pension. What will happen later, if my son won’t be able [to pay for it]?” (P11_26) [subtheme: expensive service]* |
| Behavioral profile of the autistic adult | + | *No subtheme* |
|  | - | *“Recently his life got much-much worse (...), he used to be relatively open, as long as he was in secondary school. (...) Since then there has been just a downhill, (...) his daily schedule got scrambled, which means that he doesn’t go to bed but stays up all night. (...) And now he cannot go there [the daycare center] either, as he is unable to get up.” (P12_32) [subtheme: sleep problems]* |
| Decision of autistic adult | + | *No subtheme* |
|  | - | *“That is, from their [the university’s] part there were all kinds of support, the problem was not with this there, but**, actually, the problem was that he [my son] did not ask for anything. Because he has always been in resistance, he did not want others to know about it [his diagnosis].” (P11_26) [subtheme: no willingness to use it]* |

Table 3. Factors supporting vs. impeding access to services and activities: broader themes and examples from the interviews
