# Supplementary material for: “…in the middle of nowhere…” Access to, and quality of, services for autistic adults from parents’ perspectives: a qualitative study
Source: Front Psychiatry. 2024 Feb 26;15:1279094. doi: 10.3389/fpsyt.2024.1279094 (PMC10946251; doi:10.3389/fpsyt.2024.1279094)
Supplement: Supplementary file 4 [file Table_4.docx]

Supplementary Material

| Broader themes | +/- | **Example quotes** |
| --- | --- | --- |
| Active and happy life, in safety | + | *“She loves it [the daycare center] because she is occupied there, there is always something to do. (...) If she does not go there for a week or two, then she misses it a lot; that is, she loves going there.” (P2_23) [subtheme: meaningful and regular activities]* |
|  | - | *“Towards the end I could see very well that he didn’t like to go [to work]. He was tired, run-down, tormented. He kept standing all day. (...) He said that he had been doing the washing up all day long.” (P10_26) [subtheme: psychological well-being]* |
| Cooperation and communication | + | *“I’d like to have a patient, helpful and determined boss for him, one who knows what he wants, who is able tell it and waits for the young man understanding it. (...) [who] allows enough liberty for him to make decisions in many things, to take care of himself, but when they see a problem, they* *are* *able to intervene, too.” (P9_25) [subtheme: communication between adults and professionals]* |
|  | - | *“It would have been good to be in touch all the time [with his boss at his sheltered employment]. The [boss] said I did not inquire. That is true (...), but I myself thought that in such a [sensitive] situation the employer should send a signal to the parent if any problem arises. But nothing like that happened. (P10_26) [subtheme: cooperation between professionals and parents]* |
| Expectations on professional work | + | *“They don’t have to have a qualification in autism, they should just sense what the child needs.” (P4_23) [subtheme: personalized support]* |
|  | - | *“There isn’t any development here, anymore; activity sessions, or so to say, individual therapy or activity sessions do not exist [in the day care center].” (P4_23) [subtheme: detectable development of autistic adult]* |
| Expectations on staff | + | *“Well,* *I think I am satisfied. As I see, they have the necessary qualification, the have their humanity,* *patience; practically, they have everything.” (P12_32) [subtheme: qualifications, competences, and skills of staff]* |
|  | - | *“I know that there are immensely too few professionals, and I know that they are awfully overloaded.” (P11_26) [subtheme: number of professionals]* |
| Practical expectations | + | *“(...) We can apply for supervision also in the summer holiday, and this is, too, a big help.” (P4_23) [subtheme: service during the summer holidays]* |
|  | - | *“I wouldn’t take him [to a daycare center], because there are too many in a group there.” (P5_28) [subtheme: appropriate client group size]* |

Table 4. Parental evaluation of services/activities: broader themes and examples from the interviews
